# S1PR2 Signaling in the Lung: Understanding Its Role in Health and Disease

**DOI:** 10.3390/cells15010010

**Published:** 2025-12-20

**Authors:** Alison W. Ha, Joe G. N. Garcia, Steven M. Dudek

**Affiliations:** 1 Division of Pulmonary, Critical Care, Sleep and Allergy, Department of Medicine, University of Illinois Chicago, Chicago, IL 60612, USA; aha6@uic.edu; 2 Department of Molecular Medicine, University of Florida Scripps Research Institute, Jupiter, FL 33458, USA; jgn.garcia@ufl.edu

**Keywords:** sphingosine-1-phosphate receptor 2, lung endothelium, lung epithelium, acute respiratory distress syndrome, pulmonary fibrosis, asthma

## Abstract

**Highlights:**

**What are the main findings?**

- S1PR2 exhibits distinct cell-type specific expression patterns in the lung, including endothelial, epithelial, immune, fibroblasts, and smooth muscle cells, each contributing to unique functional outcomes in tissue homeostasis and injury responses.
- S1PR2 plays mechanistically important roles across multiple lung diseases, influencing processes such as vascular permeability, inflammation, airway reactivity, and fibrotic remodeling.

**What are the implications of the main findings?**

- S1PR2 signaling is complex and context-dependent, with its effect varying by cell type, microenvironment, and disease state.
- Therapeutic targeting of S1PR2 remains underdeveloped, highlighting the need for deeper investigation into cell-specific and disease-specific interventions and more selective modulators.

**Abstract:**

Sphingosine-1-phosphate receptors (S1PRs) are a family of G protein-coupled transmembrane proteins that play essential roles across nearly all organ systems, including the regulation of pulmonary physiology and immune responses. Expressed across diverse lung cell types, S1PRs mediate critical biological processes such as vascular barrier integrity, immune cell trafficking, and inflammation. While the signaling pathways and physiological functions of S1PR1 and S1PR3 have been extensively characterized, the role of S1PR2 remains less clearly defined and context-dependent. In this review, we summarize current knowledge on S1PR2 signaling within major pulmonary cell populations and explore its contribution to lung homeostasis and disease. By synthesizing evidence from molecular, cellular and in vivo studies, this review aims to summarize the current understanding of S1PR2 signaling across major pulmonary cell populations and its roles in lung homeostasis and disease. The findings of this study could help develop new strategies for treating pulmonary disorders and other diseases by targeting S1PR2.

## 1. Introduction

Sphingosine-1-phosphate (S1P) is a bioactive lipid mediator that regulates diverse physiological and pathological processes [1], including vascular development [2], immune cell trafficking [3], and inflammation [4]. S1P is generated from sphingosine [4], a major lipid found in eukaryotic cell membranes [5]. The molecular structure of S1P comprises a sphingoid backbone, a fatty acid tail, and a phosphorylated head group [6]. The biosynthesis of S1P is catalyzed through the phosphorylation of sphingosine by two isoenzyme kinases, sphingosine kinase 1 and 2 (SphK1/2) [7]. Once formed, S1P levels are regulated by two primary pathways: a reversible pathway mediated by S1P phosphatases, which hydrolyze S1P back to sphingosine, and an irreversible degradation pathway, where S1P lyase cleaves S1P into hexadecanal and phosphoethanolamine [8]. This irreversible degradation is critical for controlling intracellular S1P levels [9].

In the circulation, S1P levels are tightly regulated, with lymph concentrations of approximately 100 nM and plasma concentrations of approximately 0.1μM-1 μM [10]. The majority of circulating S1P is bound to albumin or apolipoprotein M-containing high-density lipoproteins [11]. These chaperone proteins play a crucial role in stabilizing S1P, facilitating its transport as both a paracrine and autocrine signaling molecule, and protecting it from enzymatic degradation [12].

A key feature of S1P biology is its ability to function extracellularly through a process termed ‘inside-out signaling’ [13]. In this mechanism, intracellularly synthesized S1P is actively transported across the cell membrane by specialized S1P transporters, such as members of the spinster homolog 2 (Spns2) and ATP-binding cassette (ABC) transporter families [14]. These transporters facilitate the release of S1P into the extracellular space, where it then can bind and activate a family of five G-protein coupled receptors (GPCRs), denoted as S1P receptor 1-5 (S1PR1-5) [15], eliciting a signaling cascade that regulates numerous cellular functions, including vascular function [16], cell–cell adhesion [17], cell migration [18], and cell survival [19]. The primary effects of S1P signaling result from this extracellular S1P binding to surface receptors expressed on nearby cells (i.e., autocrine/paracrine effects), as well as distant cells reached via circulating blood and lymph. It is unclear what relative percentage of these S1P signaling effects occurs in an autocrine/paracrine fashion compared to more distant events. Via these receptor-mediated pathways, S1P exerts diverse effects across nearly all organ systems. In the lung, S1P signaling is critical for pulmonary vascular stability, immune homeostasis, and inflammatory responses [20]. Disruptions in S1P metabolism or receptor signaling have been implicated in multiple pulmonary diseases, including acute respiratory distress syndrome (ARDS), pulmonary fibrosis, and asthma [21]. Given the complexity and context-dependent effects of S1P signaling, its role in lung pathophysiology remains an important area of active investigation. While S1P itself and S1PR1 have been extensively studied as targets for lung disorders [22,23], the functions of S1PR2 remain comparatively underexplored despite growing evidence that it plays a critical role in pulmonary biology [24,25,26]. This review focuses on the role of S1PR2 in lung physiology and disease, with an emphasis on its regulation of vascular permeability, immune responses, and tissue remodeling across multiple pulmonary pathologies. Here, we examine mechanistic insights into S1PR2 signaling, define its context-dependent functions, and evaluate its potential as a therapeutic target in lung disease.

## 2. S1P Receptors

S1P exerts its broad biological effects primarily through S1PR1-5 [15]. These receptors, belonging to the endothelial differentiation gene (Edg) family, display distinct yet overlapping expression patterns across various cell types and tissues. This varied distribution allows S1P to regulate diverse cellular and physiological processes [13]. Most cells express at least one S1P receptor, while many express multiple subtypes [27], allowing both paracrine and autocrine manners of signaling [1]. Each receptor subtype couples to specific heterotrimeric G-proteins, leading to activation of unique downstream pathways [28]; however, some functional redundancy and crosstalk exist between these receptors [29]. Additionally, S1P engagement can stimulate receptor internalization from the plasma membrane to the cytoplasm, regulating receptor availability, desensitization, and proteasomal degradation, further adding to the complexities of S1P signaling [30,31]. Beyond its receptor-mediated actions, S1P also exerts receptor-independent, intracellular functions, regulating processes such as inflammation [32], vascular permeability [33], and calcium mobilization [34]. Together, these complementary mechanisms highlight the importance of S1P signaling in both homeostasis and disease. While all five receptors contribute to the versatility of S1P signaling, each subtype displays distinct signaling characteristics that is defined by its tissue distribution, G-protein coupling, and downstream pathways. S1P has high affinity for all five S1PRs, though its functional affinity for each receptor is influenced by both structural variations in the receptor binding pockets and by the differential expression patterns of these receptors across cell types [35]. This section will provide an overview of the major biological functions of S1PR1-5.

### 2.1. S1PR1/EDG-1

S1PR1/EDG-1 couples to the Gαi family of heterotrimeric G-protein [36] and is highly expressed in the lung, particularly in endothelial cells, but also in smooth muscle and immune cells, where it plays an essential role in vascular development [37] and immune regulation [38]. Like most GPCRs, S1PR1 is a cell surface receptor that signals at the plasma membrane; however, it is also localized to intracellular compartments, including vesicles, the cytoplasm, and the nucleus [39], allowing spatially distinct signaling functions. Upon activation by S1P, S1PR1 undergoes conformational changes that activate multiple signaling cascades, notably Ras/ERK, PI3K/Akt, and Rac1 [40]. Through these pathways, S1PR1 regulates fundamental cellular processes such as survival, proliferation, and migration [41]. One of the most prominent roles of S1PR1 is in immune cell trafficking, directing lymphocyte egress from lymphoid tissues to maintain immune surveillance [42]. It also influences adaptive immunity by promoting the differentiation of T-helper 17 cells [43], supporting immune homeostasis and the regulation of inflammatory responses in the lung [43]. Beyond immune modulation, S1PR1 is arguably the most critical regulator of vascular barrier integrity [44,45]. Through Rac1- and Rho-mediated cytoskeletal remodeling, S1PR1 signals the assembly and stabilization of adherens and tight junctions between endothelial cells [2], reinforcing barrier integrity and limiting vascular leak [46]. Taken together, these roles highlight S1PR1 as a critical mediator of vascular and immune regulation, functions that have been extensively detailed in the literature [22,47,48].

### 2.2. S1PR2/EDG-5 

**Figure 1:**
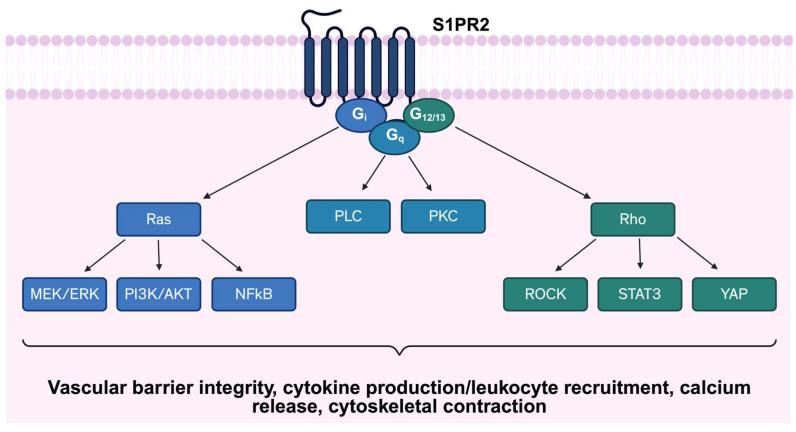
Schematic representation of S1PR2 signaling pathway. S1RP2 couples to multiple G-proteins, including Gαi, Gα12/13, and Gαq, initiating diverse downstream signaling cascades. Abbreviations: S1PR2: Sphingosine-1-phosphate receptor 2; PLC: Phospholipase C; PKC: Protein Kinase C; MEK/ERK: Mitogen-activated protein kinase/Extracellular signal-regulated kinase; PI3K: Phosphoinositide 3-Kinase; AKT: Protein Kinase B; NFκB: Nuclear factor-kappa B; ROCK: Rho associated protein kinase; STAT3: Signal transducer and activator of transcription 3; YAP: Yes-associated protein.

S1PR2/EDG-5 couples to multiple G-proteins, including Gαi, Gα12/13, and Gαq [31], as illustrated in Figure 1, and is localized to both the plasma membrane and cytoplasm [39]. S1PR2 has lower expression in lung ECs compared to S1PR1 [49]; however, it is found highly expressed in alveolar epithelial cells, as well as alveolar macrophages, fibroblasts [26], and smooth muscle cells [50]. While S1PR2 shares downstream signaling pathways with S1PR1, such as PI3K and ERK [51], S1PR2 often exerts opposing effects. S1PR2 activation inhibits cell migration [52], contrasting the promigratory role of S1PR1 [38], underscoring the functional diversity of S1P-S1PR signaling. In the vascular system, S1PR2 exerts context-dependent effects on barrier integrity [53]. Activation of Gα12/13 promotes cytoskeletal contraction and stress fiber formation, destabilizing endothelial junctions and increasing vascular permeability [54,55]. Conversely, S1PR2 signaling can also reduce phosphorylation of Akt and endothelial nitric oxide (eNOS), thereby stabilizing adherens junctions and protecting against vascular barrier disruption [56]. Beyond the vasculature, S1PR2 contributes to epidermal homeostasis and barrier maintenance in the skin [57]. Together, these findings highlight the complex and context-dependent functions of S1PR2 across different tissue types. Beyond its role in barrier regulation, S1PR2 also influences inflammatory processes. Its expression in immune cells modulates cytokine production, linking S1PR2 activity to both tissue homeostasis and response to pathological stimuli [58,59]. Through its diverse signaling interactions, S1PR2 plays a crucial role in maintaining homeostasis and responding to pathological stimuli. In the sections that follow, we discuss its specific contributions to lung homeostasis and pathogenesis in greater detail.

### 2.3. S1PR3/EDG-3 

S1PR3/EDG-3 couples to Gαi, Gαq, and Gα12/13 [36] and is localized to the plasma membrane [60] and is highly expressed in endothelial cells [49], smooth muscle cells [50], fibroblasts [61], and macrophages [62], where it can activate a broad array of signaling pathways, including Ras, ERK, PI3K/Akt, PLC, and Rho [63]. Through this diverse signaling, S1PR3 influences angiogenesis [64], immune responses [65], and vascular permeability [60]. Functionally, S1PR3 overlaps with both S1PR1 and S1PR2. Similar to S1PR1, S1PR3 contributes to angiogenesis and endothelial function, though in a redundant manner [66]. Loss of S1PR1 alone results in severe vascular defects, whereas combined deletion of S1PR1 and S1PR3 does not significantly worsen these abnormalities [66], suggesting that S1PR3 plays a supportive role rather than an essential one in vascular development. Beyond this redundancy, S1PR3 has distinct physiological and pathological functions, particularly in the lung and immune system. In the pulmonary endothelium, receptor activation drives Rho-mediated cytoskeletal rearrangements that can disrupt barrier integrity and increase vascular permeability [67]. S1PR3 is also strongly implicated in fibrotic processes, where it promotes epithelial-to-mesenchymal transition and fibroblast activation, contributing to extracellular matrix deposition and tissue remodeling [53]. Within the immune system, S1PR3 plays a complex, context-dependent role by regulating leukocyte rolling and adhesion [68], enhancing myeloid cell oxidative killing of microbial pathogens [69], and promoting dendritic cell maturation and Th1-mediated responses [65], thereby shaping both innate and adaptive immune responses. S1PR3’s contributions to the inflammatory cascade, however, are more complex, as inhibition of S1PR3 suppresses NLPR3 inflammasome activation in sepsis models [70] and reduces NF-kB-driven cytokine production [71], suggesting a potential role in limiting excessive immune activation. Conversely, S1PR3 signaling can also enhance macrophage and neutrophil recruitment, thereby amplifying innate immune responses during infection and inflammation [65]. Taken together, these findings highlight S1PR3 as a receptor with overlapping, but also distinct functions, compared to other S1PRs. S1PR3 involvement in vascular regulation, fibrosis, and immunity is discussed in detail elsewhere [39,72]. 

### 2.4. S1PR4/EDG-6 

S1PR4/EDG-6 is expressed predominantly in myeloid and lymphoid cells [73] and couples to Gαi and Gα12/13 [74]. Downstream, S1PR4 activates ERK, PLC, and RhoA pathways, with the potential to modulate a range of immune responses [75,76]. A highly prominent S1PR4 function is the regulation of neutrophil recruitment [73]. Genetic studies, including large-scale meta-analyses, identified a missense variant in *S1PR4* to be associated with lower circulating neutrophil counts [77], demonstrating a key role in innate immunity. Beyond neutrophils, S1PR4 is expressed in dendritic cells, where it contributes to both differentiation [78] and migration [79], as well as in macrophages to modulate cytokine production through Akt signaling [80]. S1PR4 also plays a role in hematopoiesis by promoting megakaryocyte differentiation [81], linking S1P signaling to platelet formation. While the inflammatory functions of S1PR4 continue to be elucidated, its broader roles in immune modulation have been detailed elsewhere [79]. Aside from S1PR4’s role in immune cells, recent studies have identified S1PR4 expression in brain microvascular endothelial cells, contributing to blood–brain barrier integrity [82], with the role of S1PR4 in the lung less well-characterized. 

### 2.5. S1PR5/EDG-8 

S1PR5/EDG-8 displays a more restricted expression profile compared to the other S1P receptors, being enriched in oligodendrocytes, skin, and natural killer cells [39]. S1PR5 couples to Gαi and Gα12/13 [74] and regulates processes such as migration, survival, and barrier stabilization [83]. Within the immune system, S1PR5 is upregulated during natural-killer (NK) cell maturation and is essential for its migration [84]. Recent studies have further expanded its immunological role, showing that S1PR5 contributes to T-cell migration and regulates the differentiation of tissue-resident memory T-cells [85], a subset critical for long-term immune protection. Within the central nervous system (CNS), S1PR5 plays a role in vascular and glial biology. S1PR5 is highly expressed in brain capillaries, where it contributes to the maintenance of blood–brain barrier integrity [86]. In parallel, S1PR5 supports oligodendrocyte survival [87], highlighting its relevance to myelination and CNS homeostasis. Beyond these structural functions, S1PR5 also exerts immunomodulatory effects within the brain by dampening endothelial activation via suppression of VCAM-1 and ICAM-1 expression, thereby limiting leukocyte adhesion and infiltration across the brain endothelium [87]. S1PR5 protects the CNS from excessive immune response, signifying its role as both a barrier stabilizer and immune regulator.

## 3. S1PR2 in Lung Cells

Despite contributing to multiple aspects of pulmonary biology, S1PR2 effects are context-dependent and vary across different lung cell types [88,89]. Increasing evidence implicates S1PR2 in diverse critical homeostatic processes, including barrier regulation [55,56], immune modulation [26], and fibrotic remodeling [61]. However, the outcomes of S1PR2 signaling remain incompletely understood. To better understand these complexities, this review will focus on its signaling mechanisms and functional consequences in key lung cell populations.

### 3.1. Endothelial Cells 

The pulmonary endothelium forms a critical interface between the bloodstream and lung tissue [90]. Lung endothelial cells (ECs) not only facilitate gas exchange, but also maintain the integrity of the vascular barrier, playing an essential role in regulating the movement of fluids, solutes, and immune cells across the lung [91]. While ECs express S1PR1, S1PR2, and S1PR3 [92], S1PR2 exerts a complex and context-dependent effect on EC function. Activation of S1PR2 is known to couple with the Rho/ROCK pathway, driving actin stress fiber formation and destabilization of adherens junctions [54]. These cytoskeletal changes increase endothelial permeability [54] and impair cell migration [92], leading to barrier disruption. Paradoxically, S1PR2 can also exert barrier protective effects under certain conditions. Specifically, S1PR2 ligation and activation reduces the phosphorylation of AKT and eNOS, thereby lowering nitric oxide (NO) production and attenuating vascular leak [56]. This dual behavior highlights the importance of cellular and environmental context in determining S1PR2-mediated outcomes. In addition to barrier regulation, S1PR2 modulates endothelial inflammatory signaling. Activation of S1RP2 upregulates p38 MAPK and NF-κB pathways, promoting cytokine production and leukocyte recruitment [92]. These pro-inflammatory pathways are especially relevant in pathological conditions such as acute lung injury (ALI), where dysregulated endothelial activation exacerbates vascular dysfunction [93]. Emerging evidence also links S1PR2 to vascular aging and senescence with levels of S1PR2 markedly increased in cultured senescent ECs and in lesion regions of atherosclerotic endothelium [[94]]. Studies in aged rodent models have demonstrated that S1PR2 expression is upregulated in pulmonary ECs of aged rats and correlates with a decline in key endothelial functions, including reduced EC migration, impaired tubule formation, and slower wound healing [95]. Silencing S1PR2 in these aged ECs restores these cellular functions, highlighting its potential as a therapeutic target in age-associated endothelial decline [95]. Together, these findings position S1PR2 as a diverse regulator of pulmonary endothelial biology, exerting both disruptive and protective effects.

### 3.2. Epithelial Cells 

The lung epithelium is essential for maintaining barrier integrity, facilitating gas exchange, and orchestrating host defense [96]. Within this heterogeneous cell population, S1PR2 is expressed in both airway and alveolar epithelial cells [97], where it plays a context-dependent role in modulating epithelial responses to injury and inflammation. In the airway epithelium, S1PR2 functions as a potent pro-inflammatory mediator stimulating STAT3 [97] and NF-κB signaling [97,98], driving the transcription of pro-inflammatory genes such as IL-8 [98], a key chemokine involved in neutrophil recruitment and activation. Through these mechanisms, S1PR2 contributes to airway inflammation and is implicated in respiratory diseases such as asthma and chronic obstructive pulmonary disease (COPD) [25,98]. In contrast to its inflammatory role in the airways, S1PR2 plays a reparative role in the alveolar epithelium [89]. In alveolar Type II (ATII) cells, which serve as progenitors for alveolar maintenance and regeneration, S1PR2 signaling is required for their differentiation into alveolar type I (ATI) cells [89]. This process is regulated by the Yes-associated protein (YAP) pathway, positioning S1PR2 as a critical upstream modulator of alveolar repair [89]. However, the influence of S1PR2 in the alveoli is not exclusively protective. Under certain pathological conditions, receptor activation can trigger epithelial-to-mesenchymal transition (EMT) through RhoA and phosphorylated SMAD3 signaling [99]. This shift towards a mesenchymal phenotype contributes to fibrotic remodeling and disease progression. Collectively, these findings highlight the context-specific and complex nature of S1PR2 signaling in the pulmonary epithelium, where it may support tissue repair or contribute to chronic inflammation and fibrosis.

### 3.3. Immune Cells

Immune cells in the lung are critical for maintaining tissue homeostasis and initiating rapid responses to pathogens and environmental insults [100]. S1PR2 has emerged as an important regulator of immune cell function in a complex manner, exerting both protective and pathological responses. Mast cells, central effectors of inflammatory responses in the lung, express high levels of S1PR2 [101]. Receptor activation enhances mast cell degranulation, leading to the release of histamine, as well as pro-inflammatory cytokines and chemokines such as CCL2, IL-6, and TNF-α [101]. These mediators amplify inflammation by recruiting additional immune cells, including T lymphocytes [102]. Notably, pharmacological inhibition of S1PR2 with the antagonist JTE-013 significantly attenuates this pro-inflammatory mediator release following antigen stimulation [101], suggesting therapeutic potential for targeting S1PR2 in allergic airway disease. Macrophages, which are essential for innate defense and tissue remodeling, also express high levels of S1PR2 [103]. Unlike its pro-migratory effects in lymphocytes and mast cells, S1PR2 signaling in macrophages restricts cell migration [26] through RhoA-dependent regulation of cytoskeletal dynamics [26,54]. Moreover, S1PR2 activation impairs macrophage phagocytic capacity [103], potentially limiting effective clearance of pathogens and resolution of inflammation. Deletion of S1PR2 in hematopoietic cells promotes type 2 immune responses, in part through increased IL-33 expression and expansion of type 2 immune cell populations [24]. Beyond an influence on migration and immune regulation, S1PR2 also promotes profibrotic macrophage signaling [26], upregulating IL-13 production, a key driver of tissue remodeling and enhancing downstream phosphorylation of STAT6 along with increased expression of IL-13 regulated genes such as *Fizz1* and *Arg1* [26]. Taken together, these findings reveal the diverse role of S1PR2 in modulating immune cell behavior within the lung. From amplifying mast-cell-driven inflammation and T-cell recruitment to impairing macrophage-mediated clearance and promoting fibrotic signaling, S1PR2 functions as a key immunomodulatory receptor with broad implications for pulmonary health and disease. 

### 3.4. SMC

Airway smooth muscle cells (SMCs) are central regulators of pulmonary physiology, coordinating the contraction and relaxation of the airway wall to control airflow and resistance [104]. While essential for normal respiratory physiology, SMCs also contribute to pathological airway remodeling in diseases such as asthma [105]. Among S1P receptors, S1PR2 and S1PR3 are most abundantly expressed in the airways, with S1PR2 playing a particularly prominent role in modulating SMC behavior [50]. Activation of S1PR2 in airway SMCs drives cytoskeletal reorganization to enhance motility and contractility, effects that are significantly attenuated by pharmacological inhibition with JTE-013 [104,106]. These functional changes are supported by S1PR2-driven calcium mobilization, a key trigger for smooth muscle contraction, and contribute to airway hyperresponsiveness, a defining feature of asthma pathophysiology [105]. Mechanistically, S1PR2 signaling activates STAT3, leading to downstream dephosphorylation of YAP and subsequent induction of Notch3, a pathway implicated in SMC proliferation [107]. The relevance of these pathways extends beyond asthma. In pulmonary arterial hypertension (PAH), S1PR2-mediated signaling drives excessive SMC proliferation and vascular remodeling, processes that elevate vascular resistance and arterial pressure [108]. Taken together, these findings demonstrate S1PR2 as a critical regulator of airway smooth muscle function.

### 3.5. Fibroblasts 

Lung fibroblasts are a diverse population of mesenchymal cells that preserve alveolar structure and synthesize components of the extracellular matrix (ECM), thereby maintaining the mechanical support and structural integrity of the lung [109]. S1PR2 signaling plays a central role in driving the profibrotic phenotype of the lung fibroblasts. Receptor activation enhances ECM synthesis [61] and promotes fibroblast-to-myofibroblast differentiation, as evidenced by increased expression of α-smooth muscle actin [110]. Additionally, S1PR2 activation increases myofibroblast proliferation, further amplifying the fibrotic response [61]. Together, these findings underscore S1PR2 as a central driver of fibroblast activation and ECM remodeling in the lung.

Collectively, studies across diverse pulmonary cell types demonstrate that S1PR2 signaling exerts context-dependent effects, regulating processes from vascular barrier regulation and inflammation to tissue repair and fibrotic remodeling. These findings are summarized in Table 1, which highlights some of the cell-specific roles and downstream consequences of S1PR2 activation. Building on these mechanistic insights, the following section examines how S1PR2 contributes to the pathogenesis of multiple pulmonary diseases.

## 4. S1PR2 in Lung Disease

### 4.1. ARDS/Sepsis 

Sepsis, a life-threatening condition arising from a dysregulated host response to infection, remains a major cause of morbidity and mortality in intensive care units (ICUs) [111]. One of the most severe consequences of sepsis is the development of ARDS, a condition characterized by diffuse alveolar damage, excessive inflammation, and disruption of the pulmonary microvascular barrier [112]. Loss of both endothelial and epithelial integrity plays a central role in the pathogenesis of ARDS, driving pulmonary edema, impaired gas exchange, and respiratory failure [113]. Recent studies identify S1PR2 as a key mediator of sepsis-induced lung injury, particularly through its effects on vascular integrity and inflammatory signaling. In a cecal ligation and puncture model, S1PR2 expression was significantly upregulated in the lung, while pharmacological inhibition with JTE-013 restored the expression of critical junctional proteins, such as ZO-1, VE-cadherin, and occludin [114], thereby improving vascular integrity. Similarly, in lipopolysaccharide (LPS)-induced sepsis, mice deficient in *S1pr2* (*S1pr2^−/−^*) exhibited reduced vascular permeability and preserved endothelial barrier function compared to wild-type controls [88,92,115]. Beyond an impact in the endothelium, S1PR2 also amplifies inflammatory signaling in sepsis. In macrophages, increased S1PR2 expression is positively correlated with mitochondrial fragmentation and dysfunction, a process associated with poor clinical outcomes [116]. Both genetic deletion and pharmacologic inhibition of S1PR2 reduced inflammation, including reduced expression of ICAM-1 and VCAM-1 [114], key mediators of leukocyte recruitment and vascular inflammation [117], and reduced plasma levels of IL-6 and MCP-1 [92]. These changes were accompanied by decreased circulating neutrophils and monocytes [92]. Interestingly, loss of S1PR2 not only suppressed pathological inflammation, but may also promote reparative immunity. Specifically, *S1pr2* deletion increased macrophage-derived IL-33 production, an enhancer of lung Type 2 response [24], and expanded group 2 innate lymphoid cells, M2 macrophages, and eosinophils [24], immune populations associated with tissue repair and resolution of injury [118,119]. While S1PR2 is largely implicated in barrier disruption and inflammatory injury during sepsis-induced ARDS, its functions are not exclusively pathogenic. In a model of *Pseudomonas aeruginosa* infection, S1PR2 promoted the transition of ATII cells to ATI cells, an essential process for alveolar repair [89]. This dual role suggests S1PR2 may contribute both to lung injury and to epithelial repair, likely dictated by disease context. 

### 4.2. Pulmonary Fibrosis 

Idiopathic pulmonary fibrosis (IPF) is a progressive interstitial lung disease characterized by excessive ECM deposition, fibroblast proliferation, and lung tissue remodeling, ultimately leading to respiratory failure [26]. A defining feature of IPF pathophysiology is chronic epithelial cell injury and turnover, which drives epithelial dysfunction and activates molecular mediators that drive EMT, fibrocyte recruitment, and fibroblast-to-myofibroblast differentiation [120]. Together, these processes contribute to persistent fibrosis and disease progression. Sphingolipid signaling has emerged as a critical regulator of pro-fibrotic signaling pathways, particularly through their modulation of pro-fibrotic molecules such as TGF-β [121], with S1PR2 gaining recognition as a pathogenic driver. In murine models, S1PR2-expressing cells accumulate within fibrotic lesions following bleomycin-induced injury, as demonstrated by *S1pr2^LacZ/+^* reporter mice [26]. Genetic deletion of *S1pr2* attenuated pulmonary fibrosis, reducing PDGFRα positive fibroblast expansion and decreasing fibronectin and collagen 1a1 mRNA expression compared to wild type controls [26]. Notably, the protective effect of *S1pr2* deficiency extended beyond fibroblast activation. *S1pr2^−/−^* mice exhibited reduced inflammatory cell infiltration and decreased protein concentrations in bronchoalveolar lavage fluid (BALF) following bleomycin exposure [26]. Transcriptomic analyses of BALF cells from *S1pr2*^−/−^ mice further revealed downregulation of genes encoding pro-fibrotic cytokines, chemokines, and markers of alternatively activated (M2) macrophages, highlighting the contribution of S1PR2 to fibrosis-associated inflammation [26]. Mechanistically, S1PR2 signaling enhances the production of IL-13, a cytokine strongly implicated in fibroblast activation and tissue remodeling [26]. The pathogenic role of S1PR2 in fibrosis is further supported by pharmacological studies. Inhibition of S1PR2 with antagonists mitigated bleomycin-induced pulmonary fibrosis, reducing collagen deposition, inflammatory cell recruitment, and expression of pro-inflammatory mediators, including IL-4, IL-5, IFN-Y, and TNF-α [26,122]. JTE-013 also downregulated mitochondrial fusion and fission proteins, thereby attenuating oxidative stress levels in fibrotic lungs [122]. More recently, the novel S1PR2 antagonist, GLPG2938, has demonstrated robust antifibrotic activity in preclinical models [123], reinforcing the therapeutic potential of S1PR2. Collectively, these findings highlight the pathogenic role of S1PR2 in IPF and suggest that targeting this receptor may provide a novel approach for the treatment of fibrotic lung disease. 

### 4.3. Asthma 

Asthma is a chronic inflammatory disease characterized by airway obstruction, hyperresponsiveness, and structural remodeling [124]. One of the prominent features of asthma is type 2 airway inflammation, which involves the recruitment of eosinophils, mast cells, basophils, neutrophils, monocytes, and macrophages into the airways [125]. These immune responses drive the production of pro-inflammatory cytokines and mediators that exacerbate airway dysfunction [125]. Recent studies have highlighted a central role for S1P-S1PR2 signaling in modulating asthmatic inflammation and airway remodeling. In murine models of ovalbumin (OVA)-induced asthma, pharmacological inhibition of S1PR2 with JTE-013 significantly reduced levels of key inflammatory cytokines, including IL-1, IL-4, IL-5, as well as serum IgE levels in BALF [126]. Pre-treatment with JTE-013 prior to antigen challenge attenuated inflammatory cell infiltration in the airways [126]. In vitro studies further support this, as inhibition of S1PR2 has been shown to reduce IL-8 release in BEAS-2B airway epithelial cells [98]. Moreover, stimulation with S1P upregulated CCL3 expression, a chemokine that is significantly upregulated in asthmatic patients [127], in airway epithelium through S1PR2 [97]. This effect, mediated by NF-kB and STAT3 transcriptional pathways, is mitigated by JTE-013 [97]. Beyond immune regulation, S1PR2 signaling further contributes to the pathophysiology of asthma by promoting airway hyperresponsiveness, intracellular calcium sensitization, and airway smooth muscle cell proliferation [105]. In vivo, pharmacological inhibition of S1PR in OVA-challenged mice reduced goblet cell proliferation and collagen deposition [126]. Emerging data also implicates S1PR2 in the regulation of autophagy during asthma progression [126]. Inhibition of S1PR2 with JTE-013 reduced OVA-induced expression of autophagy markers Beclin1 and LC3 [126], suggesting S1PR2 may influence disease severity through regulation of autophagic processes. Taken together, these findings suggest that S1PR2 is a central mediator to airway inflammation, hyperresponsiveness, and structural remodeling. Targeting S1PR2 signaling could be a promising therapeutic approach for attenuating disease progression and improving outcomes in asthmatic patients.

### 4.4. Additional Lung Diseases 

**Figure 2:**
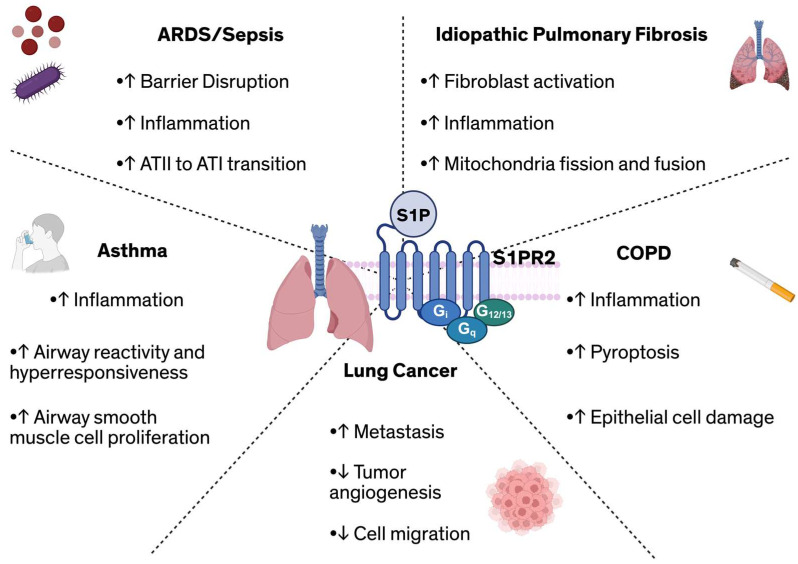
Overview of S1PR2-mediated effects across diverse pulmonary diseases, including its role in regulating ARDS/sepsis, fibrosis, asthma, lung cancer, and COPD. Abbreviations: ARDS: Acute respiratory distress syndrome; ATI/ATII: Alveolar type I/II cell; COPD: Chronic obstructive pulmonary disease; S1P: Sphingosine-1-phosphate; S1PR2: Sphingosine-1-phosphate receptor 2.

Beyond established roles in ARDS, IPF, and asthma, S1PR2 has also been implicated in the pathogenesis of other pulmonary diseases, including lung cancer, and COPD. In lung cancer, S1PR2 exhibits context-dependent effects, functioning as both as a tumor-suppressor and a tumor-promotor. For example, S1PR2 signaling has been shown to inhibit tumor angiogenesis and cell migration [128], thereby restricting tumor growth, with *S1pr2^−/−^* mice exhibiting a two-fold increase in tumor microvessels compared to WT controls, supporting a protective role for S1PR2 in limiting tumor vascularization [128]. Conversely, other studies suggest that S1P-S1PR2 signaling promotes lung metastasis by downregulating breast cancer metastasis suppressor 1 (Brms1), a key metastasis inhibitor, thereby facilitating tumor progression [129]. In COPD, S1PR2 expression is elevated in alveolar macrophages isolated from patients [130]. Experimental models of cigarette smoke-induced COPD further demonstrate that exposure to cigarette smoke extract (CSE) upregulates S1PR2 expression in bronchial epithelial cells [25]. Functionally, knockdown of S1PR2 in these cells improved bronchial epithelial cell morphology following CSE exposure, suggesting a protective effect against smoke-induced epithelial damage. Moreover, overexpression of S1PR2 has been associated with increased transcription and translation of key pyroptosis markers, including NLRP3, IL-1b, IL-18, and caspase-1 [25]. This promotes the release of pro-inflammatory cytokines and exacerbates airway inflammation. The diverse contributions of S1PR2 across the various pulmonary pathologies discussed in this review are summarized in Figure 2.

## 5. Conclusions

Taken together, the studies reviewed here demonstrate the diverse and disease-specific roles of S1PR2 across various pulmonary cell types and pathophysiologic states (Table 1, Figure 2). This work highlights that S1PR2 signaling regulates fundamental physiological and pathological processes, including maintaining endothelial and epithelial barrier integrity in ARDS [89,114], promoting fibrotic remodeling in IPF [26], and driving airway inflammation and hyperresponsiveness in asthma and COPD [126]. Importantly, these studies reveal that S1PR2 can exert both pathogenetic and protective effects.

Despite significant advances, important gaps remain in our understanding of S1PR2 biology within the lung. Current evidence suggests that S1PR2 exerts context-dependent and cell-type specific effects, influencing inflammation, fibrosis, and barrier regulation. For example, in endothelial cells S1PR2 activation can either disrupt or protect vascular integrity depending on the signaling environment [54,56], yet the molecular determinants that dictate these outcomes remain poorly defined. A major limitation in the field is our incomplete understanding of how S1PR2 functions across the heterogenous endothelial population of the lung [131]. Endothelial cells from microvascular, macrovascular, and capillary compartments possess distinct transcriptional and functional identities [131], suggesting that S1PR2 signaling may produce spatially restricted or opposing effects on vascular permeability, repair, and leukocyte trafficking. Similarly, in epithelial cells, S1PR2 drives pro-inflammatory and pro-fibrotic signaling yet also contributes to alveolar repair under certain conditions [89,98]. In immune cells, S1PR2 enhances mast cell activation, yet it also impairs macrophage migration and phagocytic function [26,101,103] further underscoring its complexity. Future studies are needed to define the upstream regulators and downstream effectors of S1RP2 signaling in the lung, and to determine how S1PR2 interacts with other S1P receptors to coordinate intercellular communication across the diverse lung cell populations. These types of studies will help clarify how S1PR2 influences the balance between injury and repair in the lung.

Therapeutically, S1PR2 remains an underdeveloped target. Despite growing recognition of its diverse role in multiple lung pathologies, there are currently no S1PR2-specific modulators approved for clinical use [132]. In contrast, several modulators of S1PR1 are already approved for the clinical treatment of immune-mediated and neurological diseases, with several additional compounds in various stages of clinical development [133]. These compounds function by inducing receptor internalization, ubiquitination, and proteasomal degradation, thereby reducing lymphocyte egress and dampening inflammatory responses [134]. Most of the S1PR1-targeted modulators that have been developed for clinical use exhibit some degree of cross-reactivity with other S1P receptor subtypes, accounting for some of their adverse and other effects [133]; however, they generally have little or no affinity for S1PR2 [134]. This notable difference underscores a major gap in translational research surrounding S1PR2. Given its dual role in both promoting and resolving inflammation and its influence on vascular and epithelial barrier function, a deeper understanding of S1PR2 signaling dynamics will be critical for identifying therapeutic strategies. Emerging evidence suggests inflammatory conditions associated with disease can modulate S1PR1 trafficking. Cytokine signaling and respiratory viral infections upregulate the lymphocyte activation marker, CD69, which interacts with S1PR1 to promote receptor internalization and subsequent lysosomal degradation [135]. Whether similar mechanisms regulate S1PR2 remains unclear, highlighting the need for further studies into the context-dependent recycling and degradation pathways. Advancing our knowledge in S1PR2 biology holds promise for the development of novel therapies aimed at mitigating lung injury and restoring homeostasis in a range of pulmonary diseases.

## Figures and Tables

**Table 1:** Summary of S1PR2-Mediated Signaling and Cellular Responses in Pulmonary Cells.

Cell Type		Associated Signaling Molecules	Cellular Function	References
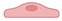	Endothelial	Rho/ROCK, AKT, MAPK, NFkB	Vascular barrier integrity, cytokine production, leukocyte recruitment, reduced cell migration	[54,56,92,95]
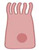	Airway Epithelium	STAT3, NFkB	Cytokine and chemokine recruitment	[97,98]
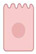	Alveolar Epithelium	YAP, RhoA, SMAD3	Alveolar repair, EMT	[89,99]
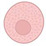	Mast	STAT3	Mast cell degranulation, recruitment of inflammatory mediators and immune cells	[101,102]
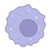	Macrophage	RhoA, STAT6	Restriction of cell migration and phagocytic activity, promotes profibrotic macrophage signaling	[24,26,54,103]
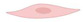	Smooth muscle	STAT3, NOTCH3, YAP	Increased cell motility, contractility, and proliferation	[104,106,107,108]
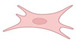	Fibroblast	PI3K/AKT	Increased ECM synthesis, increased fibroblast to myofibroblast transition, increased myofibroblast proliferation	[109,110]

Abbreviations: RhoA: Ras homolog family member A; ROCK: Rho associated protein kinase; AKT: Protein Kinase B; MAPK: Mitogen-Activated Protein Kinase; NFκB: Nuclear factor-kappa B; YAP: Yes-associated protein; SMAD3: Mothers Against Decapentaplegic Homolog 3; EMT: Epithelial-to-mesenchymal transition; STAT3: Signal transducer and activator of transcription 3; STAT6: Signal transducer and activator of transcription 6; Notch3: Neurogenic locus notch homolog protein 3; PI3K: Phosphoinositide 3-Kinase; ECM: Extracellular matrix.

## Data Availability

No new data were created or analyzed in this study.

## Abbreviations

The following abbreviations are used in this manuscript:

ABC	ATP-binding cassette
ALI	Acute lung injury
ARDS	Acute respiratory distress syndrome
ATI	Alveolar Type I
ATII	Alveolar Type II
BALF	Bronchoalveolar lavage fluid
Brms1	Breast cancer metastasis suppressor 1
CNS	Central nervous system
COPD	Chronic obstructive pulmonary disease
CSE	Cigarette smoke extract
ECs	Endothelial cells
ECM	Extracellular matrix
Edg	Endothelial differentiation gene
EMT	Epithelial-to-mesenchymal transition
eNOS	Endothelial nitric oxide
GPCR	G-protein coupled receptors
ICU	Intensive care unit
IPF	Idiopathic pulmonary fibrosis
LPS	Lipopolysaccharide
PAH	Pulmonary arterial hypertension
S1P	Sphingosine-1-phosphate
S1PR	Sphingosine-1-phosphate receptors
SMCs	Smooth muscle cells
SphK	Sphingosine kinase
Spns2	Spinster homolog 2
YAP	Yes associate protein
